# Unique transcriptomic response to sepsis is observed among patients of different age groups

**DOI:** 10.1371/journal.pone.0184159

**Published:** 2017-09-08

**Authors:** Steven L. Raymond, María Cecilia López, Henry V. Baker, Shawn D. Larson, Philip A. Efron, Timothy E. Sweeney, Purvesh Khatri, Lyle L. Moldawer, James L. Wynn

**Affiliations:** 1 Department of Surgery, University of Florida College of Medicine; Gainesville, Florida, United States of America; 2 Department of Molecular Genetics and Microbiology, University of Florida College of Medicine; Gainesville, Florida, United States of America; 3 Division of Biomedical Informatics Research, Department of Medicine, Stanford University School of Medicine; Stanford, California, United States of America; 4 Institute of Immunity, Transplantation, and Infection, Stanford University; Stanford, California, United States of America; 5 Department of Pediatrics, University of Florida College of Medicine; Gainesville, Florida, United States of America; 6 Department of Pathology, Immunology, and Laboratory Medicine, University of Florida College of Medicine; Gainesville, Florida, United States of America; University of Kansas Medical Center, UNITED STATES

## Abstract

Sepsis is a major cause of morbidity and mortality, especially at the extremes of age. To understand the human age-specific transcriptomic response to sepsis, a multi-cohort, pooled analysis was conducted on adults, children, infants, and neonates with and without sepsis. Nine public whole-blood gene expression datasets (636 patients) were employed. Age impacted the transcriptomic host response to sepsis. Gene expression from septic neonates and adults was more dissimilar whereas infants and children were more similar. Neonates showed reductions in inflammatory recognition and signaling pathways compared to all other age groups. Likewise, adults demonstrated decreased pathogen sensing, inflammation, and myeloid cell function, as compared to children. This may help to explain the increased incidence of sepsis-related organ failure and death in adults. The number of dysregulated genes in septic patients was proportional to age and significantly differed among septic adults, children, infants, and neonates. Overall, children manifested a greater transcriptomic intensity to sepsis as compared to the other age groups. The transcriptomic magnitude for adults and neonates was dramatically reduced as compared to children and infants. These findings suggest that the transcriptomic response to sepsis is age-dependent, and diagnostic and therapeutic efforts to identify and treat sepsis will have to consider age as an important variable.

## Introduction

In 2008, sepsis accounted for over 700,000 hospitalizations in the United States with a 29 percent 28-day mortality and an associated cost of $14.6 billion [1, 2]. The incidence of sepsis and sepsis-associated mortality is bimodal, with peaks occurring in neonates (particularly those born preterm) and in adults (especially the aged) [3–9]. Despite the prevalence and impact of sepsis in all age groups, our understanding of the human response to sepsis across the spectrum of age remains inadequate and cripples our ability to modify outcomes. Mechanistic studies in mice and observational studies in humans aimed at bridging this knowledge gap have demonstrated a clear impact of age on the immunologic response to sepsis [10–13].

Systems biology approaches can be used to dissect the pathophysiology of complex diseases such as sepsis. In particular, transcriptomic profiling can identify diagnostic and prognostic gene signatures, highlight novel therapeutic targets, and uncover mechanisms behind differential sepsis outcomes. However, retrospective comparisons across age groups were not previously feasible because of methodological differences among datasets. The recent development and validation of COmbat CO-Normalization Using conTrols (COCONUT), a technique that successfully considers inter–data set batch effects while remaining unbiased to the diagnosis of the diseased patients, makes comparisons of transcriptomic data across all age groups possible for the first time [14]. To determine if there is an age-specific host response to sepsis, we identified public human genome-wide expression microarray datasets containing samples of adults, children, infants, and neonates with and without sepsis, and performed a multi-cohort, pooled analysis.

## Methods

### Study approval

Since the data were deidentified and in the public domain, they were defined as ‘nonhuman research’ under Federal Code 45 CFR 46.

### Data collection

We searched the National Center for Biotechnology Information (NCBI) Gene Expression Omnibus (GEO) and the European Bioinformatics Institute (EBI) ArrayExpress for public human clinical microarray genome-wide expression studies. As previously described, datasets containing whole blood samples with collection times within 48 hours of sepsis diagnosis were utilized, as well as datasets containing whole blood samples from healthy controls [15]. We identified nine datasets containing 688 samples from 272 adults (≥ 18 years old), 141 children (>2–17 years old), 125 infants (1–24 months old), 126 neonates (0–28 days old), and 24 patients with unknown age (Accession numbers: GSE25504, EMTAB4785, GSE40396, GSE66099, GSE40586, GSE13015 GPL6106, GSE13015 GPL6947, GSE57065, GSE33341; GSE66099 includes all unique patients from GSE4607, GSE8121, GSE9692, GSE13904, GSE26378, GSE26440; Table 1). Cohort specific selection criteria for septic and control subjects are provided in Table A in S1 File. Microbiology data for bacterial, viral, and fungal infections of septic subjects are provided in Table B in S1 File.

**Table 1:** Summary of the public human genome-wide expression microarray datasets containing samples of adults, children, infants, and neonates with and without sepsis.

Accession	Age Group	Sample Count (septic:healthy)	Platform
gse25504	Neonates	93 (48:45)	Affymetrix Human Genome U219 Array
EMTAB4785	Neonates, Infants	36 (17:19)	Affymetrix Human Gene 1.0 ST Array
gse40396	Infants	30 (8:22)	Illumina HumanHT-12 V4.0 expression beadchip
gse66099	Infants, Children	235 (188:47)	Affymetrix Human Genome U133 Plus 2.0 Array
gse40586	Children, Adults	36 (18:18)	Affymetrix Human Gene 1.0 ST Array
gse13015 gpl6106	Adults	58 (48:10)	Sentrix Human-6 expression beadChip
gse13015 gpl6947	Adults	25 (15:10)	Illumina HumanHT-12 V3.0 expression beadchip
gse57065	Adults	81 (56:25)	Affymetrix Human Genome U133 Plus 2.0 Array
gse33341	Adults	94 (51:43)	Affymetrix Human Genome U133A 2.0 Array

### Data processing

Preprocessing of data included the use of up-to-date Simple Omnibus Format in Text (SOFT) files for probe-to-gene mapping. All Affymetrix data were robust multi-array average (RMA) renormalized from original cell intensity files (CEL), and probes were summarized to genes with fixed-effects meta-analysis, as previously described [15, 16]. All microarray data were co-normalized by COCONUT version 1.01, as previously described by Sweeney *et al*. [14]. In brief, COCONUT accounts for differences between datasets by assuming that healthy controls are derived from the same distributions. Here, the co-normalization of healthy controls across the age spectrum is a violation of this assumption (since some genes do change at baseline during aging), meaning that the COCONUT-co-normalized data were accurate only in terms of fold change above baseline, not necessarily in absolute expression. We thus investigated the question of how relative changes in gene expression during sepsis are affected by age.

### Statistical analysis

Prior to statistical analysis of gene expression profiles, data from the 24 subjects with unknown ages and 28 samples collected 48 hours after sepsis diagnosis were excluded from the datasets, leaving samples from 418 septic and 218 control subjects. Gene expression patterns were compared among 244 adults (159 septic, 85 healthy), 141 children (109 septic, 32 healthy), 125 infants (86 septic, 39 healthy), and 126 neonates (64 septic, 62 healthy) using an unsupervised analysis of gene expression profiles setting a coefficient of variance greater than 50%. Principal Component Analysis (PCA) was conducted using Partek^®^ Genomics Suite^®^ Version:6.6. A supervised analysis was also performed identifying genes that were significant at *p*<0.001 (F-test). Leave-one-out cross-validation was performed to compute the misclassification rate, and Monte Carlo simulations and permutation analysis were conducted to determine whether the miscalculation rate was significantly better than predicted by chance using BRB ArrayTools™ Version:4.5.1-Stable Release as described previously [13, 17, 18]. A false discovery rate (FDR)-adjusted probability of less than 0.001 (*q*) using Significance of Microarrays™ as implemented in BRB-ArrayTools™ was also performed. Once significant genes were identified, Gene Ontology™ analysis was performed using BRB ArrayTools™. Hierarchical and K-means clustering were generated using DNA-Chip Analyzer (dChip) software. Fold changes of significant genes were calculated between septic age groups and within age groups. Functional pathway analysis was performed on significant genes using Ingenuity Pathway Analysis (IPA) software™ (Ingenuity Systems, Redwood City, CA, USA). Significant pathways were determined using a–log p-value greater than 1.3 and a Z-score greater than 2 or less than -2.

## Results

### Unsupervised analysis after COCONUT

**Fig 1:**
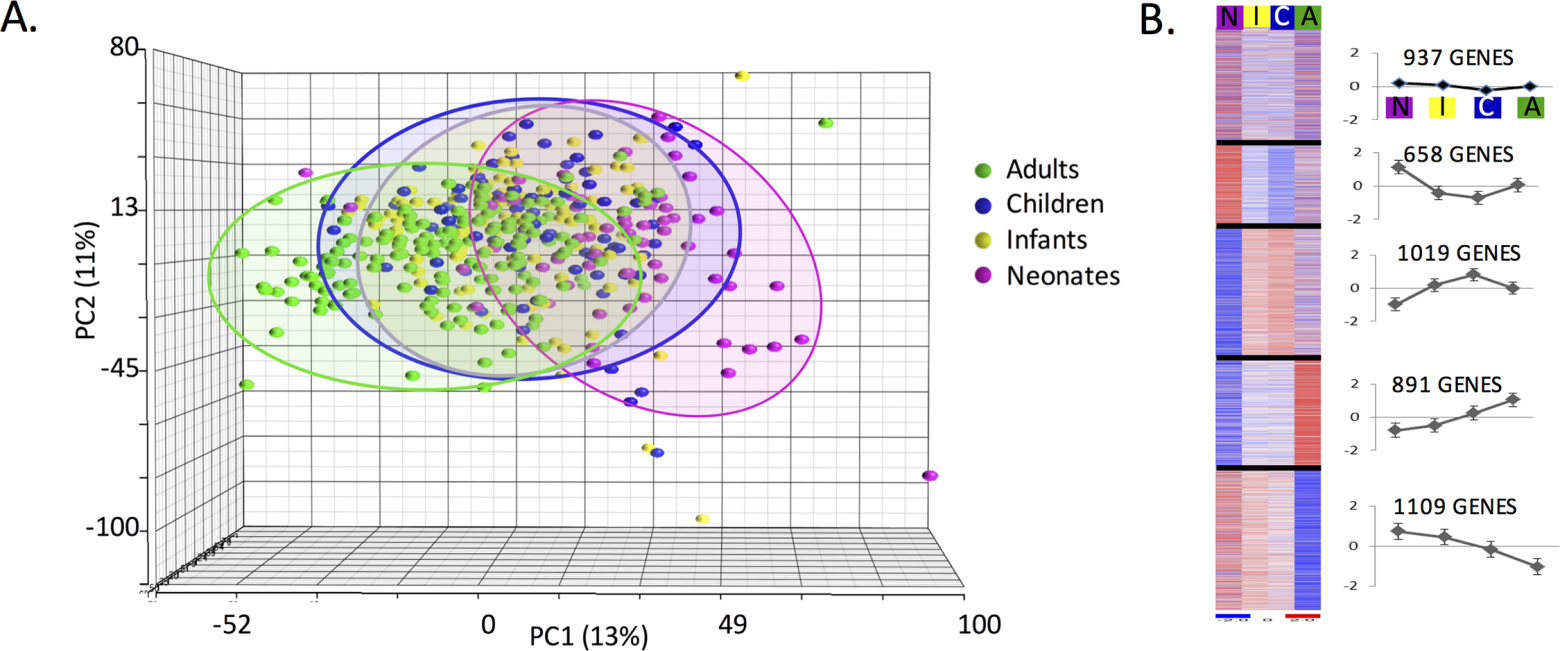
Expression profiles of patients with sepsis from all age groups. (A) Principle component analysis of the 4,535 genes whose expression differed with a coefficient of variance greater than 50% identified by unsupervised analysis. (B) Averaged arrays (f-test, 4,614 genes) with K-means clustering into five bins with mean centroid z-scores plotted for each bin and the number of genes in each bin. Error bars represent standard deviation. N-neonate, I-infant, C-children, A-adult.

Following co-normalization, an unsupervised analysis of gene expression revealed 4,535 genes whose expression differed with a coefficient of variance greater than 50%. Classifiers could not distinguish at p<0.001 between septic aged adults (≥55 years old, n = 87) versus other septic adults (18–55 years old, n = 72), so aged patients were not segregated into adult subgroups for analysis. Principal component analysis (PCA) showed that, qualitatively, the genome-wide response to sepsis across the age groups is most similar between infants and children with neonates and adults being more dissimilar (Fig 1A).

### Supervised analysis after COCONUT

The expression of 4,614 genes differed significantly among all septic age groups using a p<0.001 threshold (F-test) (Table C in S1 File). A similar number of dysregulated genes (4,264) was identified using a FDR-adjusted probability of less than 0.001 (*q*). Leave-one-out cross-validation was able to distinguish septic subjects by age group with 74% correct classifiers (p<0.001). K-means clustering into five bins for expression pattern-related sets of genes showed clear differences among age groups (Fig 1B). The averaged profiles of k-mean clustering for all bins demonstrated that the expression was markedly lower in adults and neonates compared to children and infants (Adults: 155.31, Children: 193.01, Infants: 197.32, Neonates: 127.92, Table D in S1 File). The third bin (1,019 genes) represented this overall pattern and included a number of classical inflammatory receptors including the interleukin -1 receptor 1 (*IL1R1*), *IL1R2*, *IL-4R*, *IL-6R*, colony stimulating factor 1 receptor (*CSF1R*), *CSF2R*, *i*nterferon alpha and beta receptor subunit 2 (*IFNAR2*), interferon gamma *(IFNG1)*, *IFNG2*, and the 12 member SLC family of transmembrane proteins involved in host immunity, as well as the inflammatory mediators hypoxia inducible factor 1 (*HIF1*), matrix metallopeptidase 8 (*MMP8*), *MMP9*, and the S100 family of peptides.

### Differences in canonical pathways between groups

**Fig 2:**
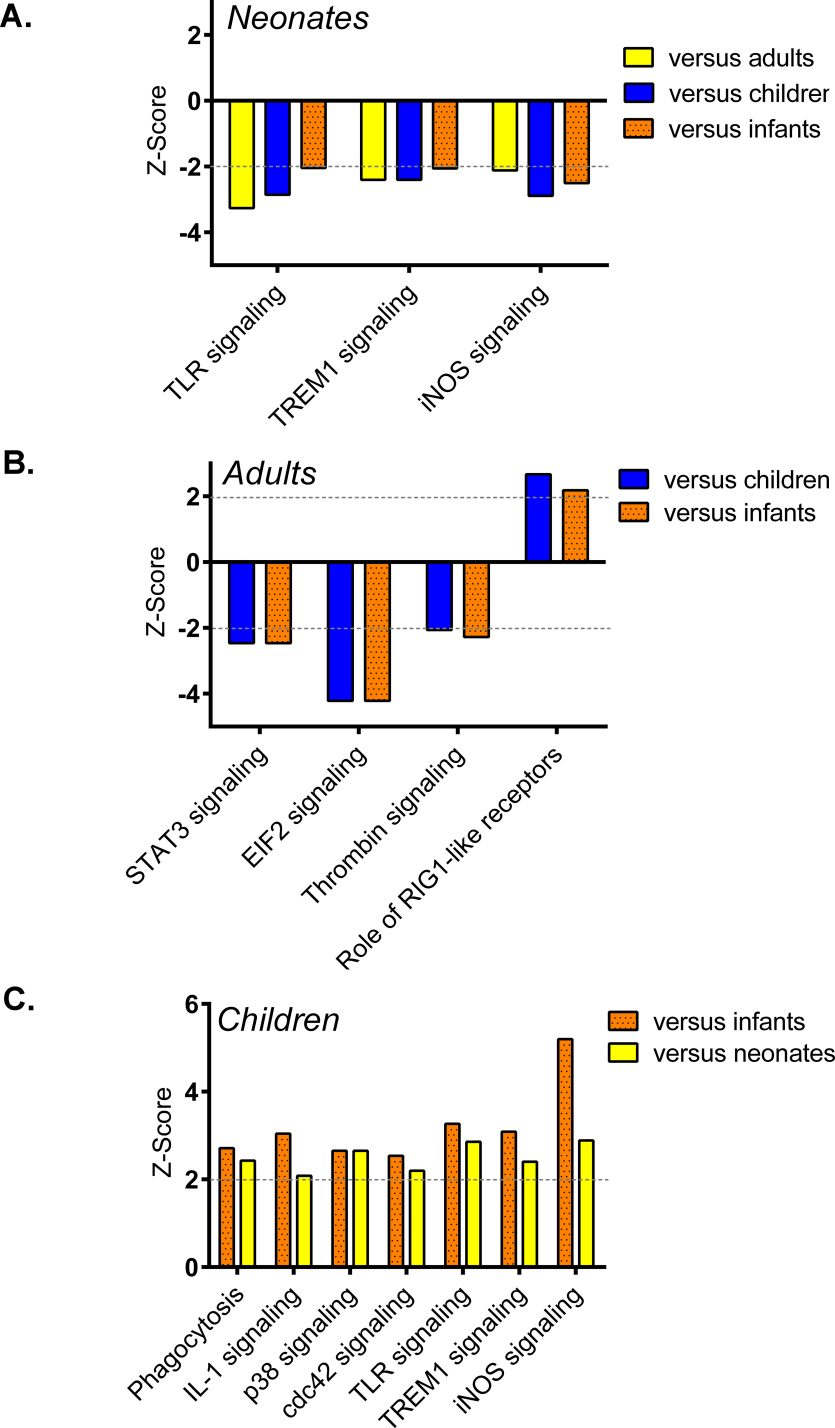
Canonical pathways analysis of 4,614 significant genes among septic adults, children, infants, and neonates. (A) Septic neonates were the only group to show significant pathway alterations (TLR, TREM1, and iNOS signaling) as compared to all other groups. (B) Compared to children or infants, adults exhibited reduced expression of genes representing STAT3, EIF2, and thrombin signaling, but increased expression of genes representing the role of RIG1-like receptors in antiviral innate. (C) A comparison of children versus infants or neonates revealed increased expression of innate immune-related pathways in children including Fcγ receptor-mediated phagocytosis in macrophages and monocytes, as well as signaling pathways for IL-1, p38, cdc42, TLR, TREM1, and iNOS.

To better understand the biologic differences, we examined the 4,614 differentially expressed genes among septic patients using Ingenuity Pathway Analysis™ (IPA). Septic neonates were the only group to show significant pathway alterations as compared to all three other groups (Table E in S1 File). Specifically, the proportion of genes representing Toll-like Receptor (TLR), Triggering Receptor Expressed On Myeloid Cells (TREM)-1, and inducible nitric oxide synthase (iNOS) signaling pathways were significantly reduced in septic neonates as compared to septic infants, children, and adults (Fig 2A). No other significant pathway differences were present in any other comparison of one group relative to all other groups.

We next identified pathways that were significantly altered in at least two of the individual group comparisons to uncover large differences in biology between groups of septic patients (Table F in S1 File). Compared to children or infants, adults exhibited reduced expression of genes representing signal transducer and activator of transcription 3 (STAT3), eukaryotic translation initiation factor 2 (EIF2), and thrombin signaling, but increased expression of genes representing the role of retinoic acid-inducible gene (RIG)1-like receptors in antiviral innate immunity (Fig 2B). A comparison of children versus infants or neonates revealed increased expression of innate immune-related pathways in children including Fcγ receptor-mediated phagocytosis in macrophages and monocytes, as well as signaling pathways for IL-1, p38, cdc42, TLR, TREM1, and iNOS (Fig 2C).

To explore variations in sepsis biology between two specific age-groups, we identified immune-related pathway variations that were unique to individual group comparisons. The comparison between children and infants revealed 49 significant and unique altered signaling pathways for key inflammatory signaling intermediates that included interferon, high mobility group box 1 (HMGB-1), tumor necrosis factor receptor 1 (TNFR1), mitogen-activated protein kinase (MAPK), granulocyte-macrophage colony-stimulating factor (GM-CSF), CD40, endothelin-1, IL-2, IL-8, IL-9, IL-17A, IL-22, and nuclear factor (NF)-κB (Table G in S1 File). In these comparisons with infants, the direction of the Z-scores showed children manifested a greater inflammatory transcriptomic response to sepsis as compared to the other age groups. Notable innate immune-related pathways that uniquely differed between adults and children included N-Formylmethionyl-leucyl-phenylalanine (fMLP) signaling in neutrophils, T Helper 2 (T_H_2) pathway, and macropinocytosis signaling. In each of these pathways, adults demonstrated a reduction in the expression of genes as compared to children. For the comparison between adults and infants, chemokine signaling and the role of nuclear factor of activated T-cells (NFAT) in regulation of the immune response were identified, and similar to results in the comparison with children, both were reduced in adults compared to infants. Comparably, expression of genes representing macrophage migration inhibitory factor (MIF)-mediated glucocorticoid regulation was increased in adults versus neonates, whereas peroxisome proliferator-activated receptor (PPAR) signaling was decreased. Unique pathway differences between neonates and children, as well as between neonates and infants, were minimal and not immune-related.

### Dysregulated genes and the intensity of the transcriptomic response to sepsis from baseline

**Fig 3:**
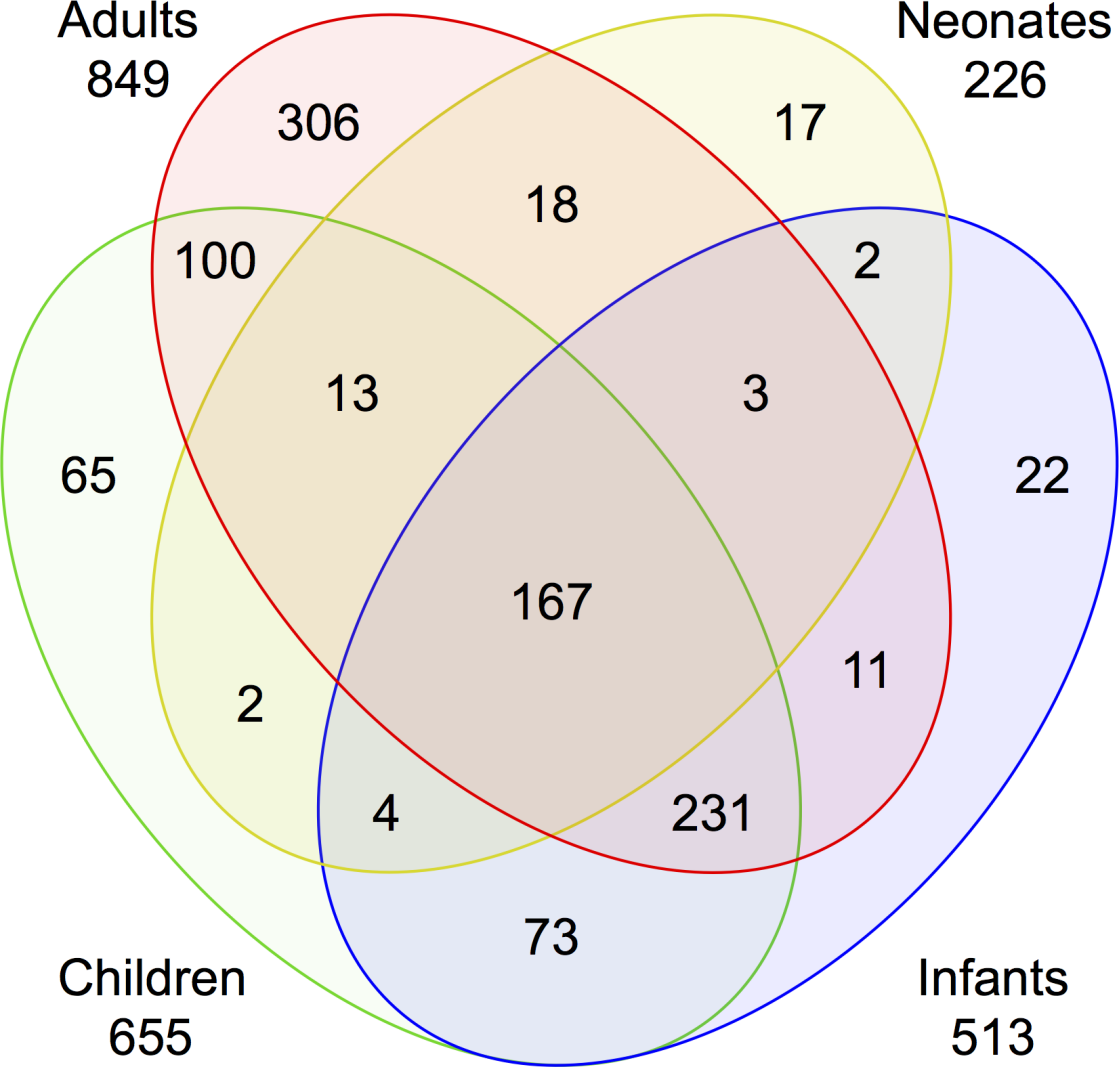
The number of dysregulated genes in septic patients from baseline is proportional to age. The number of significant (p<0.001) and differentially expressed (≥ 2-fold in either direction) genes for each age group and in each overlap between age groups. A cluster of 167 genes was identified in septic compared to healthy subjects regardless of age.

**Fig 4:**
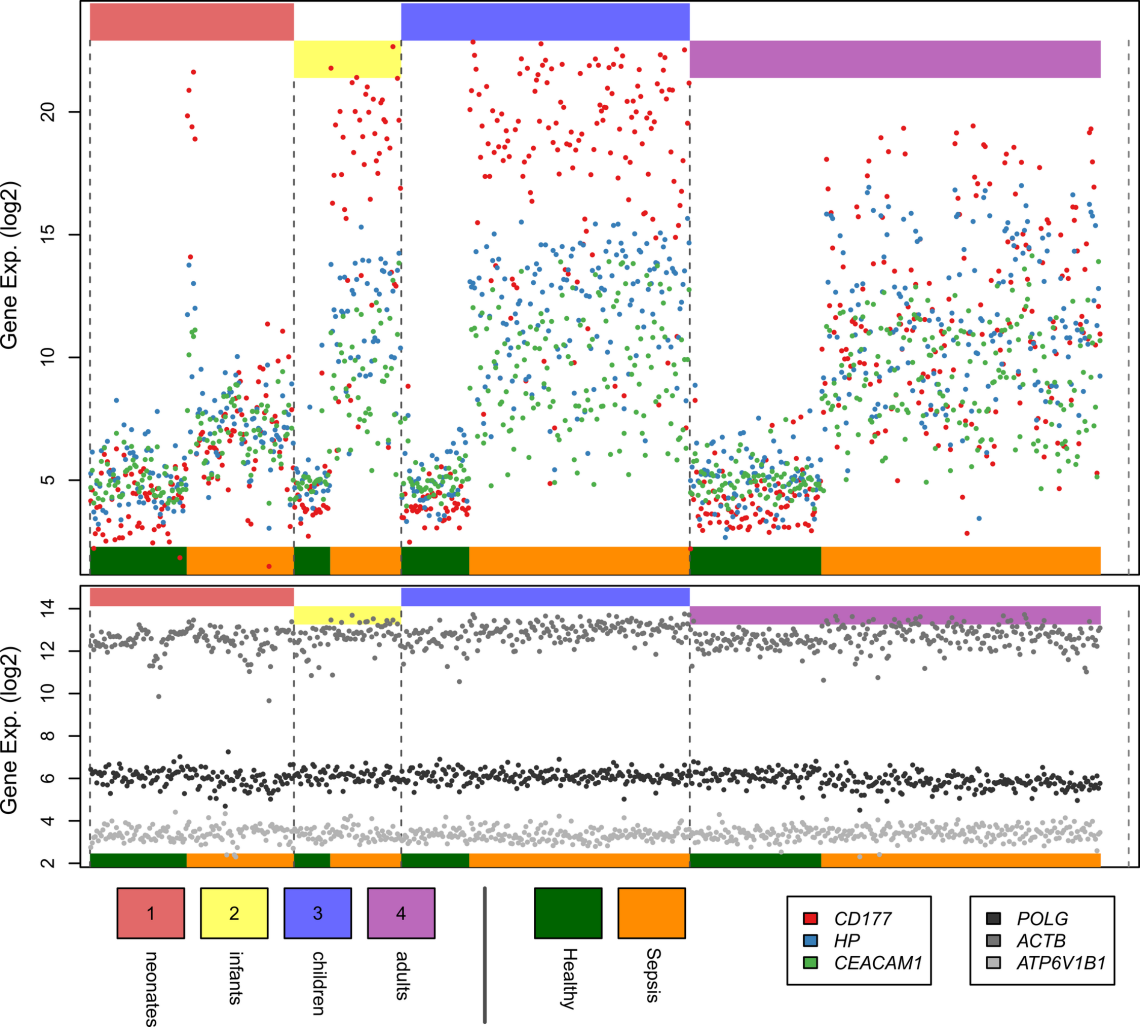
Sample-level gene expression data for COCONUT-normalized pooled sepsis data. Shown are the three genes with the greatest fold-change differences between age groups (top panel), along with three housekeeping genes (bottom panel). Top colored bars indicate age groupings; bottom bars (green and gold) show grouping of healthy and septic subjects within each age range. Within subgroups, subjects are further sorted by age. Data are shown in log_2_ scale. Exp-expression.

In *intra-age* comparisons examining the response to sepsis, the number of altered genes was proportional to age (Adults: 849, Children: 655, Infants: 513, Neonates: 226, Fig 3, Tables H-K in S1 File). A cluster of 167 genes was identified whose expression changed in septic patients as compared to healthy subjects regardless of age (Fig 3, Table L in S1 File). The magnitude of the upregulation of shared immune-related genes was markedly less in septic adults and neonates as compared to septic children and infants (Fig 4). The largest discrepancy in fold-change between groups were key components of the neutrophilic immune response to pathogens and included *CD177*, lipocalin 2, lactotransferrin, bactericidal/permeability-increasing protein, and *MMP8*.

## Discussion

This is the first comparison of the transcriptomic host response to sepsis across the spectrum of human development. Although the response across the ages was qualitatively more similar than it was different, there was a noticeable impact of age. The averaged profiles of k-mean clustering for the 4,614 differentially expressed genes demonstrated that the expression was markedly lower in adults and neonates compared to children and infants. As a specific example of this difference between groups, expression of *MMP8*, which is required for effective neutrophil (PMN) chemotaxis to lipopolysaccharide (LPS) [19], was dramatically reduced in neonates and adults relative to children and infants. This finding may explain, in part, the poor PMN chemotaxis seen in septic neonates [20, 21] and septic adults [22]. In experimental models, the absence of *MMP8* is associated with increased mortality due to increased bacterial burden [23].

Our finding that septic neonates had significant reduction of the proportion of genes representing TLR, TREM-1, and iNOS signaling pathways as compared to septic infants, children, and adults, in concert with the reduced expression of multiple cytokine receptors, are consistent with several studies that have demonstrated a reduced early inflammatory response to pro-inflammatory triggers or sepsis in neonates as compared to infants, children, and adults [10–13, 24]. Similarly, the reduction in immune pathways we describe in septic adults would be expected to lead to a decrease in early myeloid cell activation and mirrors our murine findings of the host response to sepsis across the age-spectrum [12]. Interestingly, septic children exhibited a greater transcriptomic response as compared to the other three age groups, particularly for genes representing pathways involved in pathogen sensing, inflammation, myeloid cell function, and the role of the Rho GTPase cdc42 in maintaining the endothelial barrier [25]. In line with these findings, we previously reported increased gene expression for pathogen recognition, NF-κB, and TREM-1 signaling in toddlers with septic shock as compared to infants [11]. Taken together, these findings may help explain the reduced incidence of cardiovascular and renal dysfunction in children with sepsis (6–7, 9, 24–25) and offers a unique perspective on why adults and neonates manifest greater sepsis mortality compared to infants and children [3–9].

While we and others have identified diagnostic transcripts to discern disease from health in a single age group [14, 26–28], our identification of a shared set of 167 genes across all age groups suggests measurement of a specific subset of these transcripts might, in turn, have utility in all age groups for predictive enrichment to accompany clinical trials, as well as diagnostic or prognostic testing for sepsis [15, 27, 29]. Of note, we found a striking reduction in the transcriptomic magnitude among the shared 167 dysregulated genes in adults and neonates compared to children and infants. Genes with the largest discrepancies in fold-change were key components of the neutrophilic immune response to pathogens. The relatively decreased expression seen with sepsis in adults and neonates for these neutrophil-related genes could reflect the presence of different circulating neutrophil populations with different functional capabilities that might contribute to an increased mortality risk [30, 31] and potentially be amenable to therapeutic modification.

There are potential limitations inherent to a study of this kind. First, a multi-cohort analysis of the transcriptomic response among patients with sepsis from different age groups could be affected by clinical and microbiological differences among the cohorts. Clinical criteria and microbiologic variables for the individual cohorts with available data are provided in Tables A and B in S1 File. The definition of sepsis among age groups was largely similar despite the lack of consensus definition in neonates [32]. Although the overall distribution of gram positive, gram negative, viral, and fungal infections was comparable between age groups, whole blood transcriptomic studies may not reveal pathogen-specific signatures [33]. Importantly, each age group was composed of multiple individual cohorts, which substantially increased the clinical heterogeneity present within each group [34]. Thus, the findings appear to be broadly applicable to age rather than to cohort differences.

Another potential limitation of the study is that the included cohorts utilized whole blood for transcriptomic evaluations, which can be influenced by the leukocyte representation; however, each age group had a minimum of 32 healthy and 64 septic patients, which should accurately represent the leukocyte milieu each age group manifests. Although COCONUT co-normalization provides fold changes that are valid in terms of a change in sepsis versus baseline across age groups, the absolute values in practice may differ because the fold-change multiples may be starting from different absolute locations due to baseline expression differences during aging. However, there is still considerable knowledge to be gained by looking at this relative gene differential expression in terms of pathway amplification and dysregulation, since gene expression fold change from baseline may have a greater physiologic relevance than absolute change. Despite these potential limitations, the findings have strong biological relevance and expand our understanding of the differences in the host response to sepsis across age groups.

In conclusion, a unique transcriptomic response to sepsis is observed among patients of different age groups. Neonates manifest reductions in critical inflammatory recognition and signaling pathways as compared to all other age groups, whereas adults have decreased pathogen sensing, inflammation, and myeloid cell function as compared to children. These findings may help to explain the increased incidence of sepsis related organ failure and death among adults and neonates. Taken together, this study supports the need for novel age-specific diagnostic and therapeutic efforts to reduce the devastating short- and long-term impact of sepsis.

## Supporting information

S1 File Table A in S1 File. Cohort specific selection criteria for septic and control subjects. Table B in S1 File. Cohort specific microbiology data for bacterial, viral, and fungal infections of septic subjects. Table C in S1 File. Expression data of genes that differed significantly among all septic age groups. Table D in S1 File. Averaged profiles of k-mean clustering for each septic age group. Table E in S1 File. Predicted differences in canonical pathways among septic age groups. Table F in S1 File. Immune-related pathways with variations in at least two of the individual group comparisons. Table G in S1 File. Immune-related pathways with variations unique to individual group comparisons. Table H in S1 File. Expression data of genes that differed significantly in response to sepsis among adults. Table I in S1 File. Expression data of genes that differed significantly in response to sepsis among children. Table J in S1 File. Expression data of genes that differed significantly in response to sepsis among infants. Table K in S1 File. Expression data of genes that differed significantly in response to sepsis among neonates. Table L in S1 File. Expression data of a common cluster of genes that changed response to sepsis regardless of age group.
(XLSX)
